# Focal eosinophilic infiltration of the liver, benign or malignant?

**DOI:** 10.1002/ccr3.4448

**Published:** 2021-08-16

**Authors:** Wouter Claeys, Anke Delie, Peter Smeets, Marie‐Angelique De Scheerder, Helena Degroote, Xavier Verhelst, Hans Van Vlierberghe, Anja Geerts

**Affiliations:** ^1^ Department of Gastroenterology and Hepatology Ghent University Hospital Ghent Belgium; ^2^ Department of Haematology Ghent University Hospital Ghent Belgium; ^3^ Department of Radiology Ghent University Hospital Ghent Belgium; ^4^ Department of General Internal Medicine and Infectious Diseases Ghent University Hospital Ghent Belgium

**Keywords:** eosinophilia, fasciola hepatica, focal eosinophilic infiltration, schistosoma, strongyloides

## Abstract

Focal eosinophilic infiltration (FEI) of the liver shares imaging characteristics with malignant hepatic lesions but should be suspected when concomitantly observing eosinophilia. While in itself benign, the cause of FEI should be sought and treated.

## Graphical abstract

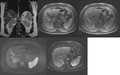

## 1 INTRODUCTION

Eosinophilia, defined as an absolute eosinophil count >500/µL, is a common occurrence and can be seen in 3%–10% of the population. Hypereosinophilia, defined as an absolute eosinophil count of >1500/µL, is however relatively rare.^1^ It has various etiologies, ranging from benign to malignant disorders. Irrespective of the cause and the severity, (hyper)eosinophilia can result in eosinophilic organ infiltration, organ damage, and abscess formation.^2^ Multiple organs can be affected, most notably lung, gastro‐intestinal (GI) system, and liver. In the liver, eosinophil accumulation is called focal eosinophilic infiltration (FEI). It is not uncommon, but discovery is mostly incidental due to increased use of imaging in often asymptomatic patients.^3^ Reported causes are parasitic infection, atopic disorders, drug hypersensitivity reactions, hypereosinophilic syndrome (HES), connective tissue diseases, and paraneoplastic eosinophilia.^3^ Upon correction of the cause of eosinophilia, the prognosis of FEI is good.^4^

Based on imaging characteristics, lesions are often misinterpreted as suspected primary hepatic neoplasms or metastases, especially in a prior context of malignancy, leading to (invasive) diagnostic testing and/or inadequate treatment.^5^ Upon encountering suspicious liver lesions in patients with eosinophilia, an extensive diagnostic workup should be performed to ensure a correct diagnosis and adequate treatment of both the liver lesion and the eosinophilia. Here, we present a case of a patient with findings of multiple liver nodules, associated with hypereosinophilia.

## 2 CASE REPORT

A 39‐year‐old male of Somali descent presented to his family physician with complaints of intermittent upper abdominal pain. Ultrasound images showed a hypo‐echogenic lesion with calcifications, and the patient was referred to the hepatology department. There was no prior personal or family history of liver disease nor alcohol abuse. Prior medical history included recurring urticaria and angioedema. Workup for atopic disorders could not find a clear cause; however, symptoms were controlled with regular systemic antihistamines. The patient worked as an office clerk. The patient had traveled to Djibouti 3 months prior. Clinical examination showed an obese man with mild abdominal tenderness in the epigastrium and right hypochondrium. No skin abnormalities could be observed. Laboratory data upon presentation showed an increase in WBC count (15,710/µL) (Ref: 3650–9300/µL) and hypereosinophilia (6237/µL) (Ref: 28–273/µL). Eosinophil count had been normal the previous year (450/µL). Furthermore, C‐reactive protein was mildly increased (6 mg/L) (Ref: <5.0 mg/L), as was ALT (54 U/L) (Ref: 7–40 U/L) and LDH (299 U/L) (Ref: 105–250 U/L). AST, GGT, and alkaline phosphatase were in the normal range. A screening panel for infectious, autoimmune, and hereditary chronic liver disease was negative. Alfa‐fetoprotein was in the normal range. IgE was elevated (334 kU/L) (Ref: 0–100 kU/L), while tryptase was in the normal range.

**FIGURE 1:**
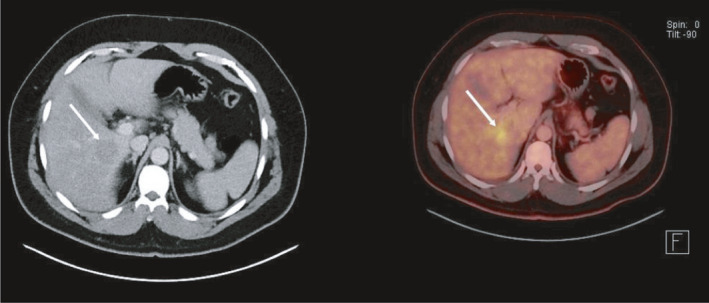
PET‐CT of liver shows a hypodense nodule in segment 5‐6 with heightened metabolic activity

**FIGURE 2:**
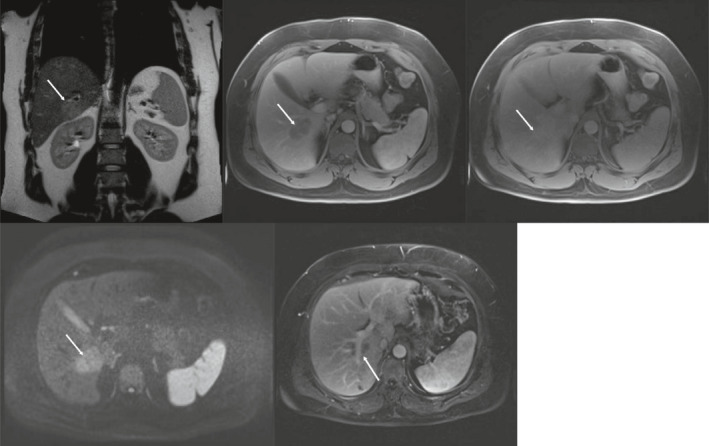
MRI findings upon initial presentation. MRI of the liver shows a large nodule in segment 5‐6 (right to left, top row). The nodule is T2 hyperintense, hypointense on T1‐weighed, contrast‐enhanced, portovenous phase images and shows enhancement in the delayed phase (right to left, bottom row). The nodule exhibits diffusion restriction and a branch of the portal vein passes through the lesion

The patient was referred to the hematology department for further evaluation of an underlying cause of the hypereosinophilia and screening for end‐organ damage. A bone marrow biopsy was performed to exclude a primary hematological cause of hypereosinophilia. The patient underwent PET‐CT to exclude malignancy and parasitic serology (toxocara Ab, schistosoma Ab [both IHA and ELISA], strongyloides Ab and fasciola Ab) was assessed. Because he complained of upper GI discomfort, an upper GI endoscopy with biopsies was performed to exclude eosinophilic infiltration. PET‐CT showed multifocal, metabolically active irregular masses anterior in the right hypochondrium, with multiple mesenterial and hilar reactive lymph nodes. In the liver, multiple metabolically active hypodense lesions were observed (see Figure 1). No bile duct dilation, portal vein occlusion, or spleen enlargement was observed. Upper GI endoscopy showed mild gastritis, helicobacter pylori positive, but without eosinophilic infiltration. Helicobacter was successfully eradicated through a 14‐day course of pantoprazole 40 mg twice daily, amoxicillin 1g twice daily and clarithromycin 500mg twice daily. Although a striking eosinophilia (24%) was observed in the bone marrow aspirate, no evidence could be found for an underlying hematological cause of peripheral hypereosinophilia on pathological, cytogenetic, and PCR studies. Follow‐up MRI showed multiple liver lesions in segment 5‐6‐7, the biggest of which was situated in segment 5‐6. Lesions were T2 hyperintense, T1 hypointense in the portovenous phase and exhibited enhancement in the delayed phase. The largest lesion exhibited diffusion restriction on diffusion weighed images. Remarkably, branches of the portal vein passed through the largest lesion (see Figure 2). Serologic testing turned out to be positive for schistosoma, fasciola hepatica, and strongyloides.

**FIGURE 3:**
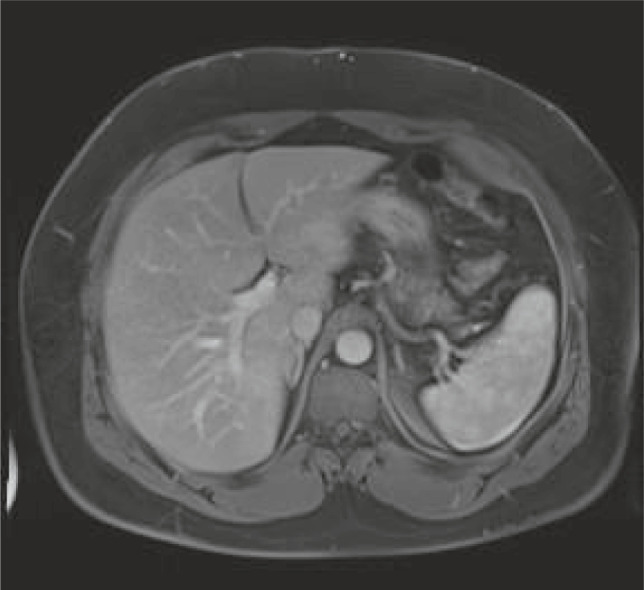
MRI findings 4 months after treatment showing complete resolution of lesions

After an episode of self‐limiting intense upper abdominal pain, the patient was admitted to the hospital. During his stay, he was treated with praziquantel (2400 mg for 3 days) and ivermectine (24 mg). This treatment has known efficacy against schistosoma and strongyloides, but limited activity against fasciola hepatica. As treatment for fasciola hepatica is not readily available in Belgium, the patient was first treated with the abovementioned drugs. Despite repeated treatment, eosinophil counts remained elevated (6510/µL), suggesting that the fasciola hepatica infection had not been resolved. The patient was additionally treated with triclabendazole 10 mg/kg. Four weeks later, absolute eosinophil counts reached near normal levels (835/µL). Control MRI of the liver 4 months after triclabendazole treatment showed complete resolution of the described lesions (see Figure 3).

## 3 DISCUSSION

The appearance of liver lesions on imaging studies with concomitant (hyper)eosinophilia is not uncommon, with reported incidence of 0.41%–0.68% of all abdominal CT scans.^3^, ^4^ Upon encountering suspicious liver lesions, the clinician should promptly assess possible malignant disease. A liver biopsy will provide the definitive answer, but this is invasive. FEI exhibits some distinctive imaging characteristics. However, despite reports of high sensitivity and specificity of imaging findings,^6^, ^7^, ^8^, ^9^, ^10^, ^11^ multiple case reports attest to the difficult differential diagnosis with malignancy when using imaging alone.^12^, ^13^, ^14^ Combining patient history, clinical presentation, laboratory values, and imaging studies is often sufficient to make a diagnosis. If doubt persists, liver biopsy still can be used to make a definitive diagnosis.

Multiple clinical and biochemical factors differentiate between malignant nodules and FEI. The mere presence of (hyper)eosinophilia is suggestive of FEI. Furthermore, indications of parasitic infection (history of eating raw food, positive serology, elevated IgE, and stay in endemic region) and (near) normal blood levels of liver enzymes and CRP are all indicative of FEI rather than malignancy.^15^

On gadolinium‐contrast MRI images, FEI typically is hypointense on T1‐weighed images, hyperintense on T2‐weighed images, and hypointense on portovenous phase images. Other features vary among investigators.^6^, ^7^, ^9^, ^10^ FEI often has ill‐defined margins as opposed to metastases.^11^ Washout in the delayed phase, characteristic for primary and metastatic liver lesions, is not often found.^9^ If the size of the lesion on unenhanced T1‐weighed images is <50% than the size on hepatobiliary phase images, this is very suggestive for FEI rather than metastasis.^5^ Interestingly, the passing of portal vein branches through these lesions, as was reported in this case (see Figure 2), has been described in FEI and suggests a benign cause rather than malignancy.^7^ CT images of FEI in the liver often show small, poorly demarcated, multifocal, hypointense lesions, most strikingly in the portal phase.^7^, ^8^, ^15^ However, CT has limited sensitivity and diagnostic accuracy compared to MRI.^16^ 18 FDG‐PET images only show increased metabolic activity in larger FEI lesions, making this technique of limited use.^8^

The clinical course of FEI of the liver is favorable, with spontaneous or treatment‐induced regression and resolution.^4^ However, FEI is a manifestation of a hypereosinophilic disorder that might necessitate treatment. Further diagnostic steps should thus be undertaken when encountering FEI/(hyper)eosinophilia.^3^, ^4^ The most common cause is parasitic infection, with Toxocara canis, Fasciola hepatica, Clonorchis sinensis, Spirometra mansonoides and Taenia solium being reported as frequent culprits.^17^ Other possible causes are drug reactions, atopic disorders, eosinophilic granulomatosis with polyangiitis, HES, and paraneoplastic eosinophilia with infiltration of the liver. Patients with GI malignancy, in particular stomach cancer, are prone to develop FEI.^4^

Further diagnostic workup should include repeat CBC to assess persistence of (hyper)eosinophilia, a basic metabolic and liver function panel, a peripheral blood smear and testing for parasitic infection. Age‐appropriate screening/oncological referral for malignancy, hematological referral and gastroenterological referral for eosinophilic GI disease should be considered on a case‐by‐case basis. For example, older patients with unexplained weight loss might be referred to an oncologist. A patient with cytopenias and splenomegaly might be referred to a hematologist. While up to 9% of patients with suspected FEI end being diagnosed with HES^3^ and up to 16% are eventually diagnosed with paraneoplastic eosinophilia,^4^ a recent retrospective analysis showed that a large portion of patients do not receive further investigation beyond whole blood cell and eosinophilic cell count.^3^ In a large portion of patients with FEI, the cause is never identified, possibly due to lack of further investigation.

In conclusion, liver lesions in patients with concomitant eosinophilia are becoming more noticeable due to increased usage of new imaging techniques. When encountering such lesions, clinicians should try to differentiate between malignant lesions and FEI that in itself has a more benign course. A combination of imaging characteristics and clinical information can suffice to make the diagnosis. If doubt persists, biopsy of the lesion can provide the answer, but this can often be avoided. Upon suspicion of FEI, one should thoroughly investigate the cause. This is often overlooked in clinical practice. The lesion itself is not typically harmful, but the cause, most often parasitic infections, HES and (gastro‐intestinal) malignancy might require treatment.

## 4 TAKE HOME MESSAGES

1. When encountering liver lesions suspect of FEI, the clinician should try to distinguish it from malignant lesions based on clinical picture, biochemistry, and imaging characteristics.
2. FEI exhibits some distinguishing imaging characteristics, but in clinical practice, this is not enough to make a diagnosis.
3. Resolution of liver lesions following treatment of hypereosinophilia is strongly suggestive of FEI.
4. Eosinophilia should be assessed, regardless of whether it has a relation with FEI.

## CONFLICT OF INTEREST

None declared.

## AUTHOR CONTRIBUTIONS

WC: wrote the manuscript and followed up on liver lesions. AD: handled hematological referral. MADS: administered and followed up on antiparasitic treatment. PS performed interpretation of MRI images. AG, XV, HV, and HD: provided supervision. AD, PS, MADS, HD, AG, XV, and HV: critically revised the manuscript. All authors read and approved the final manuscript.

## ETHICAL APPROVAL

Informed consent was obtained from the patient for the publication of this case report.

## Data Availability

The authors confirm that all relevant data are available within the article and its supplementary materials.
